# Garlic essential oil in water nanoemulsion prepared by high-power ultrasound: Properties, stability and its antibacterial mechanism against MRSA isolated from pork

**DOI:** 10.1016/j.ultsonch.2022.106201

**Published:** 2022-10-12

**Authors:** Miaomiao Liu, Yue Pan, Mingxing Feng, Wei Guo, Xin Fan, Li Feng, Junrong Huang, Yungang Cao

**Affiliations:** a School of Food Science and Engineering, and Natural Food Macromolecule Research Center, Shaanxi University of Science & Technology, Xi'an 710021, China; b Department of Life Science, Yuncheng University, Yuncheng 044000, China

**Keywords:** Garlic essential oil, High-power ultrasound, Nanoemulsion, Stability, Foodborne MRSA, Antibacterial mechanism

## Abstract

Food-borne methicillin-resistance *Staphylococcus aureus* (MRSA) has caused significant health threats and economic loss in livestock and poultry products. Garlic essential oil (GEO) is an effective antibacterial agent but presents strong instability and hydrophobicity. In this study, GEO in water nanoemulsion (GEON) with good stability was produced by emulsification technique of high-power ultrasound. Its antibacterial activity and underlying mechanism against MRSA isolated from retailed pork were investigated. Results showed that ultrasonic treatment significantly reduced the particle size of GENO from 820.3 to 215.0 nm as time increased from 0 to 10 min. Comparatively, GEON of 10 min ultrasound was more stable than other GEONs (0, 1, 5 min) during 30 d storage. It also displayed good thermal stability and relatively good ion stability (NaCl, MgCl_2_, and glucose). Antibacterial analysis showed that GEON (10 min) exhibited the best anti-MRSA activity among all GEONs, and the minimum inhibitory concentration of GEO in this nanoemulsion was 0.125 % (1.25 mg/mL). Treatment of GEON (10 min) significantly suppressed the cell proliferation of MRSA, which was mainly achieved by damaging the cell membrane as evidenced by membrane depolarization and considerable leakage of intracellular nucleic acids and protein. Laser scanning confocal microscope and scanning electron microscopy showed that treatment of GEON (10 min) significantly altered the membrane integrity and severely damaged the cellular membrane and structure. The present work illustrated that GEON produced by ultrasonic emulsification is a promising alternative to inhibit the contamination and spread of MRSA in livestock and poultry products.

## Highlights

• Garlic essential oil nanoemulsion (GEON) was prepared at different ultrasonic times.
• The particle size and PdI of GEON were reduced as time increased (0–10 min).
• GEON prepared by 10 min ultrasonication had good storage and application stability.
• GEON (10 min) showed the highest anti-MRSA activity among all GEONs.
• The anti-MRSA mechanism of GEON (10 min) was revealed.

## Graphical abstract

**Figure:**
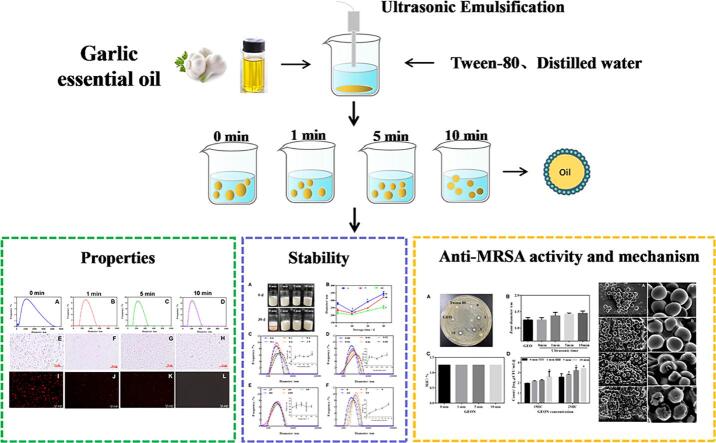

## 1 Introduction

Livestock and poultry products are important components of the diet to provide essential nutrients to human beings. They are easily contaminated by various food-borne spoilage and pathogenic bacteria during production, transportation and sales processes, which causes significant health threats and tremendous economic losses. Methicillin-resistance *Staphylococcus aureus* (MRSA) is one common pathogen that usually causes severe infections and diseases in hospital and community. However, over the past decades, MRSA has been isolated frequently in various livestock and poultry foods, including pork, beef, chicken and dairy; meanwhile, the numbers are increasing annually [1], [2], [3], [4]. MRSA possesses a large number of staphylococcal virulence factors and drug-resistance genes, which may lead to severe food poisoning, multiple drug resistance and even serious hospital outbreaks [5]. Chemical preservatives have usually been used in foods to inhibit microorganisms and prolong the shelf-life for decades, but imprudent use of them has led to the frequent occurrence of bacterial resistance and environmental pollution [6], [7]. Therefore, it is urgent to explore practical and environmentally friendly bacteriostats to control and eliminate MRSA in the food field.

Garlic (*Allium sativum* L.), an important food and medicine, has been cultivated worldwide for centuries. It is not only used as a traditional ingredient in varied delicious food, but also plays an essential role in treating headaches, bites, hypertension, atherosclerosis, intestinal worms, tumors and other diseases [8], [9]. Recently, the antibacterial activity of garlic and its derived products has attracted considerable attention. And garlic essential oil (GEO), a pale yellow oily liquid with main components of diallyl disulfide, dimethyl trisulfide, and other sulfur compounds, has been reported to exhibit noticeable antibacterial ability against *Cronobacter sakazakii*, *Staphylococcus aureus* and other bacteria [10], [11], which showed intense potential in food preservation and safety. However, the drawbacks of garlic essential oil, such as volatility, pungent flavor and low water solubility, which affect the duration of antibacterial effectiveness and the sensory quality of food [8], are the main challenges that strongly limit its development and application in foods.

Nanotechnology is one rapidly developed technology that is widely used in bioactive compound encapsulation [12]. Nanoemulsions, among various nanoencapsulation systems, are especially appropriate for essential oil encapsulation due to their nano droplet size, good stability and low turbidity [13]. It is promising in improving the utilization, stability and efficacy of essential oil, since it could encapsulate lipophilic active components into colloidal-based delivery systems and protect them from environmental factors such as high temperature, pH and oxygen. As for nanoemulsion preparation, high-power (low frequency) ultrasound is one efficient and controllable technology combining the advantages of low energy consumption, low investment cost and little equipment contamination [14]. It has been successfully used to produce nanoemulsions with a small particle size from diverse essential oil, including *thymus daenensis* oil [13], lemon oil [15] and clove oil [16]. However, the formation of garlic essential oil nanoemulsion (GEON) with low surfactant content through high-power ultrasound is still in its infancy. Moreover, nano-sized nanoemulsion generally has a raised active surface area compared to pure essential oil, which may lead to altered physical stability and antibacterial activity [17], [18]. Hence, there is a need to evaluate the stability of ultrasonically prepared GEON under different environmental conditions. Moreover, the antibacterial activities of essential oil changed variously when they convert to nanoemulsions, as contrary results were found in different oil types, bacterial species and ultrasound treatments [13], [15], [19]. Although GEO has been researched widely for its effective antibacterial activity against various microbes [10], [11], till now, few reports have concerned its nanoemulsion against foodborne MRSA and the possible mode of anti-MRSA action.

Therefore, in this study, GEONs containing 10 % GEO and low surfactant content (1 % Tween 80) were firstly formulated using ultrasonic emulsification at different treatment times (0, 1, 5, 10 min). Their properties (particle sizes, polydispersity index (PdI), zeta potential), storage and application stability and antibacterial activity against MRSA isolated from pork were evaluated. Moreover, GEON with good stability and effective anti-MRSA activity was selected and investigated for its possible mechanisms of action by determining the changes of MRSA cells growth, membrane potential, membrane integrity, extracellular nucleic acids and protein concentration, and alternations in membrane microstructures. The results will provide possibilities of new approaches to explore GEO as a natural antimicrobial additive and agent.

## 2 Materials and methods

### 2.1 Preparation of GEON

GEO was purchased from Anhui Kaibo Biological Technology Co., ltd (China). It was extracted from the garlic bulb using the steam distillation method. For coarse emulsion preparation, GEO, surfactant (Tween 80) and distilled water (weight ratio of 10:1:89) were mixed and homogenized at 18000 rpm for 120 s using a high-speed homogenizer (FA25; Shanghai Fluke Fluid Machinery Manufacturing, China) [15]. As for GEON preparation, the resulting coarse emulsion was further emulsified using a 20 kHz ultrasonic facility (SCIENTZ-ⅡD, Ningbo Xinzhi Biotechnology, China) under different ultrasonic times (0, 1, 5, 10 min) and power input of 500 W. During sonication, a 6 mm diameter titanium probe with amplitude of 220 μm was inserted into the middle of liquid, and an ice bath was necessary to maintain low temperature during the whole ultrasonication process.

### 2.2 Characterization of GEON

#### 2.2.1 Particle size, PdI and zeta potential analysis

The particle size and PdI value of GEON were analyzed by Zetasizer Nano ZS laser diffractometer (Austria Anton Pa Co., ltd.) [20]. GEON was diluted to a ratio of 1:100 with deionized water before measurement to avoid multiple scattering influences. The zeta potential of GEON was also determined using the mentioned sample dilution ratio and apparatus. Duplicate samples were measured in triplicate to ensure the repeatability of the analysis.

#### 2.2.2 Morphologic observation

The morphology of GEON was observed using the optical microscope and laser scanning confocal microscopy (LSCM) (LSM 800, Carl Zeiss, Germany), respectively. For optical microscope observation, 10 µL of prepared GEON was added on a clean glass slide, covered with a coverslip and then observed at a 100 × oil immersion lens. As for LSCM analysis, Nile red fluorescence dye was used to stain the oil phase of GEON. In brief, 0.1 % Nile red solution and GEON were mixed (1:25, v/v) and incubated at 4℃ for 20 min, shielding from light. Then 10 µL of stained GEON was placed on a glass slide, covered with a coverslip, and detected by LSCM at excitation/emission wavelengths of 543/598 nm.

### 2.3 Stability analysis of GEON

#### 2.3.1 Storage stability

All GEONs were added into tightly sealed glass tubes and stored for 30 d at 25 ± 4℃. The droplet size and visual observations were monitored at storage times of 0, 10, 20 and 30 d. Any oil layer at the bottom of the samples was considered physical instability.

#### 2.3.2 Application stability

GEON with good storage stability was further analyzed for its application stability according to the method of Zhang et al. [21] with some modifications. GEON was treated under different temperatures (20, 40, 60, 80, 100℃), different ions (0–1.0 mol/L NaCl and 0–0.08 mol/L MgCl_2_) and glucose (0–14 %, weight/volume) solutions, and then measured for the change of droplet size using Zetasizer Nano ZS laser diffractometer.

### 2.4 anti-MRSA activity analysis of GEON

#### 2.4.1 Inhibition zone determination

The antimicrobial activity of GEON against foodborne MRSA was firstly tested using the agar diffusion method. MRSA *P*-1 isolated from retailed pork was used as tested bacteria. As reported previously [22], MRSA *P*-1 had strong resistance to β-lactams antibiotics, as the MICs of penicillin and ampicillin were 1024 and 128 μg/mL, respectively. It was also insensitive to erythrocin, terramycin, acheomycin and ciprofloxacin, and the MICs were 128, 128, 32, 8 μg/mL, respectively.

The inhibition zone on the solid media was measured to evaluate the anti-MRSA activity of GEON. The circular filter paper at 6 mm in diameter was sterilized and placed on a tryptone soya agar (TSA) containing 50 µL MRSA *P*-1, whose cell concentration was approximately 10^8^ CFU/mL (OD_600 nm_ = 0.5). Afterwards, 15 µL of GEONs and 1 % Tween 80 (negative control) were added scrupulously onto the filter paper. Meanwhile, 1.5 µL of pure GEO and 13.5 µL of sterilized water were added together as control, as the proportion of GEO in the GEON was 10 %. The inhibition zones of the above agents were measured after the plates were incubated at 37℃ for 24 h.

#### 2.4.2 Minimum inhibitory concentration (MIC) determination

The MIC of GEON was investigated using the agar dilution assay. Briefly, melted TSA, GEON (or 1 % Tween solution) were aseptically transferred into 24 well plates and gently mixed. The final concentrations of GEON were 0, 3.725, 6.25 12.5, 25, 50, 100 mg/mL (0–10.0 %), namely, the final GEO concentrations were 0, 0.3725, 0.625 1.25, 2.5, 5, 10 mg/mL (0–1.0 %), respectively. When cooling, the medium was spotted with 3 μL (approximately 3 × 10^5^ CFU) of logarithmic phase bacterium and then cultured in a bacteriological incubator (37℃, 24 h). The MIC was defined as the lowest GENO concentration that inhibited the visual growth of MRSA *P*-1.

#### 2.4.3 Bactericidal ability analysis

The bactericidal assay was carried out to compare the anti-MRSA activity of four GEONs. MRSA *P*-1 at the logarithmic phase was adjusted at OD_600 nm_ = 0.5 (approximately 1 × 10^8^ CFU/mL). GEONs were added into bacterium suspension to final concentrations of 1MIC and 2MIC. Then the viable cells were measured at 2 and 4 h using the colony counting method.

### 2.5 anti-MRSA mechanism of GEON

#### 2.5.1 Growth curves analysis

Growth curves of MRSA *P*-1 under GEON treatment was analyzed based on the methodology of Liu et al. [22] with slight modification. GEON with good stability and effective anti-MRSA activity and 1 % Tween solution (negative control) were added to fresh tryptone soy broth (TSB) to final concentrations of 0-1MIC of GEON. Logarithmic phase MRSA *P*-1 was adjusted to OD_600 nm_ = 0.5 (10^8^ CFU/mL) and then inoculated (3 % bacterial load) into the prepared TSB mentioned above. Then samples were cultured at 37℃ and monitored at 600 nm for 24 h by a UV spectrophotometer (UV-2600, Shimadzu, Japan).

#### 2.5.2 Membrane potential analysis

DiBAC_4_(3) (Molecular Probes, Sigma), a membrane potential-sensitive fluorescent probe, was used for membrane potential determination [22]. MRSA *P*-1 cells were cultured to the logarithmic phase and harvested by centrifugation, and then the precipitates were washed and adjusted to OD_600 nm_ = 0.5. DiBAC_4_(3) solution was added to the cell suspensions and the final concentration was 0.1 µM. After 30 min incubation, the mixtures were added with GEON (final concentrations of 0, 1/2MIC, 1MIC, 2MIC, 4MIC) and placed at dark for 3 h. the fluorescence of each sample was determined using a fluorescence microplate reader (Thermo Scientific, Germany) at excitation/emission wavelengths of 492/515 nm.

#### 2.5.3 Extracellular nucleic acids and protein determination

The nucleic acids and protein released from MRSA *P*-1 treated with GEON were analyzed according to Moghimi et al. [13]. Logarithmic *P*-1 cells were treated with different final concentrations of GEON (0, 1/2MIC, 1MIC, 2MIC, 4MIC) for 1 and 3 h. The mixture was centrifuged and the supernate was collected for analysis. The released nucleic acids and protein were determined at 260 and 280 nm, respectively, using a spectrophotometer.

For protein determination, the amount of protein released from MRSA *P*-1 cells treated by GEON for 3 h was quantitatively analyzed using BCA protein quantitation kit (Sangon Biotech, Co., ltd.) [23]. Moreover, SDS-PAGE was performed to detect the residual soluble protein in *P*-1 cells. After being treated by GEON for 3 h, cells were centrifugally collected and resuspended with saline solution, followed by 5 min ultrasound pyrolysis in an ice water bath. The supernate containing the residual intracellular soluble protein was obtained through high-speed centrifugation. The concentration of stacking and separating gel used for SDS-PAGE were 12 % and 4 %, and the electrophoresis parameters were 80 V 40 min and 120 V 1 h, respectively.

#### 2.5.4 Membrane integrity determination

Laser scanning confocal microscopy and fluorescent dyes of SYTO9 and propidium iodide (PI) (LIVE/DEAD^@^ BacLight™ Bacterial Viability Kit, Eugene, OR) were used to observe the bacterial membrane integrity alteration of *P*-1 after GEON treatment. SYTO9 can stain all bacteria cells and glows green fluorescent, while PI only can enter bacteria through destroyed membrane, and glows red fluorescent by binding to intracellular nucleic acid. Therefore, bacteria with intact or damaged membrane will be stained as green and red fluorescent, respectively. Logarithmic *P*-1 cells (OD_600 nm_ = 0.5) were incubated with GEON (0, 1MIC, 2MIC, 4MIC) for 3 h, and then harvested by centrifugation. Cells (100 µL) were mixed with working dye solution (100 µL) and observed by LSCM after 15 min incubation.

#### 2.5.5 Scanning electron microscopy (SEM) observation

The morphology change of MRSA cells was detected by SEM (FEI Verios 460, FEI, USA). In brief, logarithmic *P*-1 cells (OD_600 nm_ = 0.5) were incubated with GEONs (0, 1MIC, 2MIC, 4MIC) for 3 h, and then centrifuged to obtain the cell pellet. After washing twice, the cell pellet was fixed with 2.5 % glutaraldehyde solution at 4℃ for 12 h. Then cells were centrifuged and dehydrated with serious water-alcohol solutions and isoamyl acetate. Afterwards, the treated cell samples were spotted onto tinfoil paper and pasted to the SEM support, sputter-coated with gold under vacuum for observing and photographing by SEM.

### 2.6 Statistical analyses

All results were conducted at least in triplicate and the differences of results were analyzed at *p* < 0.05 and *p* < 0.01 significance levels using SPSS 21.0 (SPSS Inc., Chicago, IL, USA).

## 3 Results and discussion

### 3.1 Characterization of GEON

#### 3.1.1 Partial size, PdI and zeta potential

Crude emulsion (0 min) and GEON prepared under different ultrasonic times (1, 5, 10 min) were analyzed for their average droplet size, PdI and zeta potential. As shown in Table 1, crude emulsion (control sample) produced only by high shear homogenization (18000 rpm, 120 s) showed the largest partial size of 820.3 nm. Ultra-sonication treatment had a significant effect on mean droplet size. Only 1 min of ultra-sonication treatment significantly reduced the partial size to 279.2 nm (*p* < 0.05). As time prolonged to 5 and 10 min, the mean droplet size of GEON decreased further to 235.4 and 215.0 nm, respectively. The decrease in *D_Z_* values was positively related to ultra-sonication energy delivered to emulsions. As ultrasonic time increased, the shear stress and disruptive energy applied on emulsions increased proportionally, thus leading to a continuous reduction of droplet size. Similar results have been reported previously. Nazari et al. [24] found that applying probe sonication significantly reduced the particles of garlic essential oil-loaded nanophytosomes from 161 nm (only homogenization treatment) to 115 nm. Gul et al. [19] reported that the increase of ultrasonic time and amplitudes caused a constant decrease in the mean droplet size of clove essential oil nanoemulsions. Nejatian et al. [25] also found that the mean droplet size of concentrated triglyceride nanoemulsions declined significantly as sonication time increased.

**Table 1:** Effect of ultrasonic time on the droplet size, PdI and zeta potential of GENO.

Ultrasonic time/min	Droplet size/ nm	PDI(%)	Zeta potential /mV
0	820.3 ± 34.0a	27.4 ± 2.2a	−3.1 ± 0.3a
1	279.2 ± 1.9b	22.9 ± 1.4b	−15.9 ± 0.4b
5	235.4 ± 0.7c	21.5 ± 2.0b	−16.9 ± 0.6b
10	215.0 ± 3.7d	19.6 ± 1.3b	−16.0 ± 0.6b

Note: Different lowercase letters in the same column indicate significant differences (*p* < 0.05).

The droplet sizes of GEON obtained here were comparable with or lower than nanoemulsions prepared from laurel [26] and thyme [27] essential oil (247 and 448 nm) under the same formula (10 % oil, 1 % Tween 80 and 89 % water, w/w). However, despite exposure to the longest sonication time (10 min), the droplet size of GEON (215.0 nm) in this study was still larger than one (lower than 100 nm) reported by Long et al. [20]. The difference could be mainly attributed to the higher surfactant concentration (10 %, Tween 80 mixed with Span 80) compared to our study (1 %, Tween 80). Sullivan et al. [28] also reported that emulsifier concentration is vital for nanoemulsion preparation, as 0.1 and 0.75 wt% Tween 80 in emulsions led to significantly different droplet sizes of 1 mm and 150 nm, respectively. Given the potential health concerns of synthetic surfactants, low surfactant content may be more accepted and convenient for food industry applications.

**Fig. 1:**
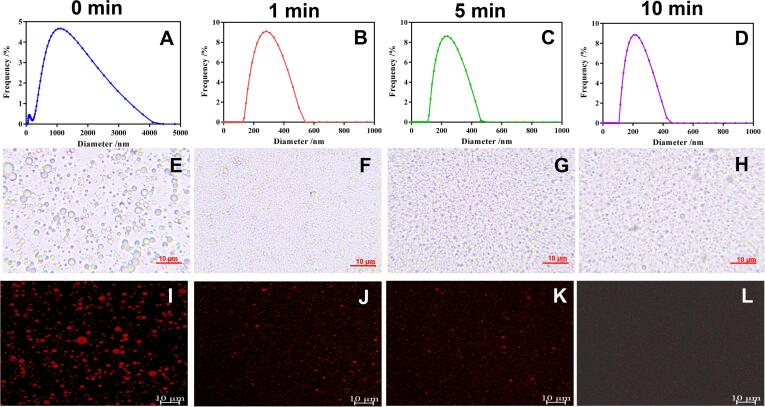
Effect of ultrasonic time on the droplet size distribution and morphology of GEON. A-D, droplet distribution of crude emulsion (0 min) and GEON prepared under different ultrasonic time (1, 5,10 min); E-H, optical microscope observation; I-L, LSCM observation.

PdI value is a dimensionless measurement for the size distribution of droplets. Generally, a small PdI value indicates a narrow size distribution, while a value higher than 0.7 represents a broad size distribution [19]. The PdI values of crude emulsion and GEONs were lower than 0.3 (Table 1), a standard that can be regarded as a monodispersed distribution. But interestingly, crude emulsion presented a bimodal and broad size distribution (Fig. 1A), whereas GEON presented a typical mono-modal droplet distribution (Fig. 1B-D). Comparatively, the ultrasonic treatment led to significantly lower PDI values than control, and a slight decrease was observed as ultrasonic time increased from 1 to 10 min, highlighting that ultrasound was effective in homogenizing the particle size of GEON.

Zeta potential represents the electrical charge of the particles and characterizes the colloidal system’s behavior, which is vital for the stability of nanoemulsion [29]. Crude emulsion and GEONs all displayed negative zeta potential, resulting from the terminal groups existing on lipids [29], [30]. Compared to crude emulsion (-3.1 mV), GEON had significantly higher absolute zeta potential values (-15.9 to −16.9 mV, Table 1), suggesting that ultrasonic treatment effectively improved the stability of nanoemulsion. It has been reported that the surface charge could predominantly affect the dispersion and coalescence of droplets in the emulsion system [31]. Thus, ultrasound may affect the stability of GEON by changing the surface charge of particles, altering the electrostatic repulsion between particles, and further influencing their aggregation [19].

However, based on the previous reports, more than 30 mV (absolute value) of zeta potential was required in colloidal systems to avoid aggregation and maintain stability [32], [33]. In this case, zeta potential of GEON were not enough for particle repulsion and stabilization, as the relatively lower values recorded here than the mentioned range. But on the other hand, Tween 80 as the nonionic surfactant could reduce the interfacial tension and formulate stable nanoemulsion by connecting the water molecular and GEO through its hydrophilic and lipophilic groups [20], [34]. Especially, the repulsion to the steric hindrance due to the polyoxyethylene chain of Tween 80 further prevents the particle coalescence. Therefore, it was speculate that the stability of GEON was simultaneously affected by zeta potential of nanoemulsion and steric effects of surfactant.

#### 3.1.2 Morphologic observation

**Fig. 2:**
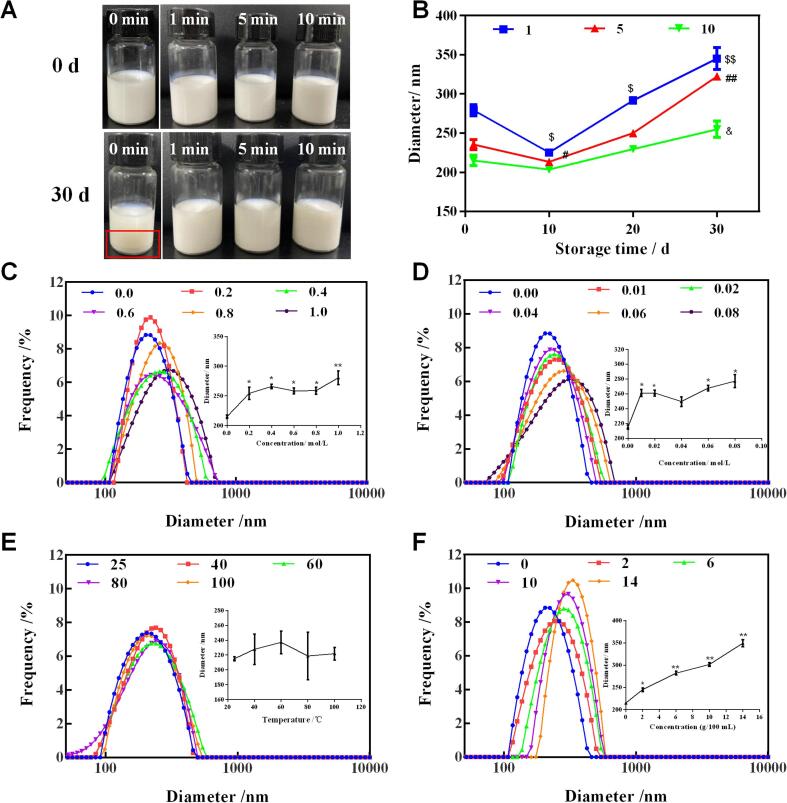
Storage stability and environmental stability of GEON. A-B, storage stability. $, # and &, *p* < 0.05; $$ and ##, *p* < 0.01. C-F, effect of NaCl, MgCl_2_, temperature and glucose on GEON stability, respectively. *, *p* < 0.05; **, *p* < 0.01.

The morphology of coarse emulsion and GEON were observed by both optical microscope and LSCM. In coarse emulsion (control, 0 min), the particle sizes distribution was nonuniform and large particles with diameter higher than 1 mm existed widely. In GEON produced by 1 min ultrasound, the size of particles reduced significantly and the uniform of particle distribution was better than control (Fig. 1F and G). In GEON with 5 min ultrasonication, the particles sizes were further reduced, and hefty particles could not be observed by optical microscope (Fig. 2G), but LSCM still found a few large particles (Fig. 2K). As ultrasonic time increased to 10 min, the particle distribution was relatively uniform, and hefty particles disappeared (Fig. 2H and L). The results were in line with the particle size distribution determined by the Zetasizer Nano ZS laser diffractometer, verifying that ultrasonic treatment could reduce the particle sizes significantly and distribute them uniformly.

### 3.2 Stability analysis of GEON

#### 3.2.1 Storage stability

Long-term storage stability is an essential precondition for the practical applications of nanoemulsions in various commercial products [25]. Therefore, coarse emulsion and GEON were investigated for their storage stability by analyzing the changes in physical appearance and droplet size in one month (Fig. 2A and B). In coarse emulsion, however, a distinct separation between GEO and aqueous phase was observed after only 1 d storage, and the oily layer (at the bottom of sealed glass tube) was pronounced at 30 d (Fig. 2A), which indicated the occurrence of demulsification. For GEON, the mean droplet size decreased firstly and then increased constantly until the end of storage (30 d), as shown in Fig. 2B. The decrease in size was probably caused by the reconstruction of emulsion to reach a stable equilibrium state, as reported by the previous reports [20]. For the increase, it was mainly caused due to “Ostwald’s ripening”, an issue in oil-in-water (O/W) emulsion that induces the size increase of large droplets at the cost of tiny droplets [35]. In the aqueous phase, oil surrounding a small particle generally showed a greater water-solubility than that surrounding a large particle. For this reason, oil can be transferred from small to large particles [36], leading to size increase and subsequent phase separation within a short period after preparation. However, despite the rise, the sizes of GEON were still in the acceptable range of nanoemulsion, and phase separation was not observed until the end of storage. Comparatively, GEON produced by 10 min ultrasonic treatment was most stable in all samples, whose droplet size maintained the minimum and changed slightly during the whole storage. The results suggested that ultrasonic treatment, especially 10 min ultrasonication, is effective in producing nanoemulsions with good storage stability.

#### 3.2.2 Application stability

Based on the long-term stability results, GEON formulated at 10 min ultrasonication was further tested under different conditions, including inorganic ions, temperature and glucose, for its application stability. The electrolyte is indispensable for various vital activities, which plays an important role in muscle contraction regulation and acid-base balance maintenance. During the production of food and beverage products, ions, such as Na^+^ and Mg^2+^, are often added to provide supplemental electrolytes for humans. Thus the influence of these ions on the properties of GEON will directly affect its application in natural food systems. Fig. 2 C and D showed the droplet size change of GEON (10 min) under different ionic strengths. As NaCl (0–1.0 mol/L) and MgCl_2_ (0–0.08 mol/L) increased, the mean droplet size increased from 215.0 to 280.5 nm and from 215.0 to 277.1 nm, respectively. However, it still presented a mono-modal size distribution, suggesting the stability of GEON when NaCl and MgCl_2_ existed. Zhang et al. [21] also found an increased particle size of emulsion stabilized by chitosan/casein complexes as NaCl addition (0–300 mM). The increase in particle size may be caused by decreased repulsion between droplets due to the electrostatic screening effect of Na^+^ and Mg^2+^, causing droplets congregation and thus the particle sizes increase. And obviously, Mg^2+^ showed a stronger electrostatic screening effect than Na^+^, since subequal droplet sizes were detected in the presence of 1.0 mol/L NaCl and 0.08 mol/L MgCl_2_.

Temperature is one of the important factors that challenge the application stability of nanoemulsion. As shown in Fig. 2E, the mean droplet size values kept around 220 nm as temperature increased (20-100℃), suggesting the strong thermal stability of GEON. Carbohydrate is another major category that provides taste and functional characteristics for food and beverage products. Glucose is one typical carbohydrate substance, and its effect on GEON was monitored. As shown in Fig. 2F, the addition of glucose produced a significant impact on the droplet size of GEON, and the size values increased from 215.0 to 349.3 nm as glucose increased from 0 to14% (weight/volume). The above results indicated that GEON was stable at high temperature, and relatively stable under NaCl, MgCl_2_, and glucose. Therefore, nanoemulsion, as a delivery system, was an effective strategy to reduce the volatilization loss and improve the stability of GEO.

### 3.3 Anti-MRSA activity analysis

**Fig. 3:**
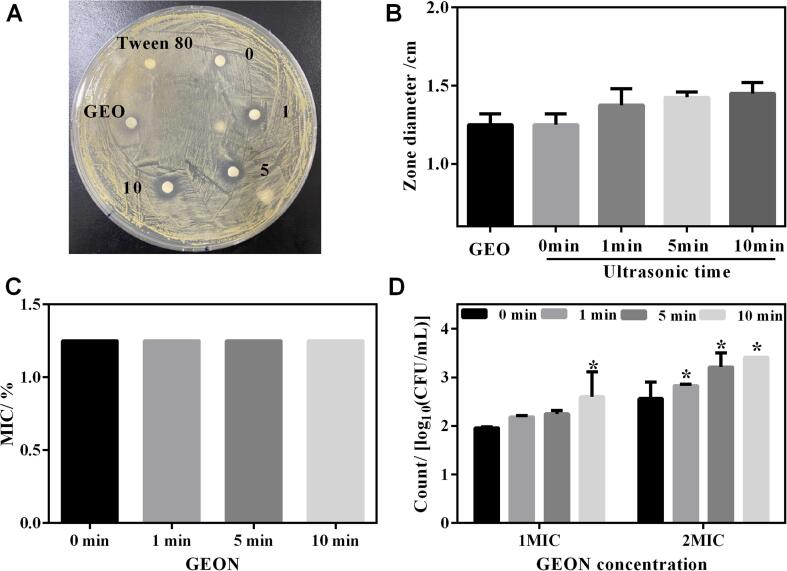
anti-MRSA activity of GEON. A-B, inhibition zone of GEON against MRSA *P*-1; C, MIC of GEON against MRSA *P*-1; D, bactericidal ability of GEON at 4 h. *, *p* < 0.05.

GEO contains many bioactive compounds such as diallyl trisulfide, diallyl disulfide, and diallyl sulfide, which are proven to have antibacterial activity against various foodborne pathogens and spoilage bacteria. The antibacterial activity of GEON against foodborne MRSA was firstly investigated using the agar disc diffusion method. As seen in Fig. 3A, coarse emulsion, GEON and pure GEO all displayed specific anti-MRSA activity with inhibition zone higher than 1.2 cm. Tween 80 (1 %) as negative control showed no activity against MRSA.

To compared, GEON showed higher anti-MRSA activity than pure GEO, while coarse emulsion had a similar inhibition zone as pure GEO (Fig. 3B), which was in accordance with the reports of other essential oils [37]. Garzoli et al. [38] reported that *Lavandula × intermedia* essential oil (LEO) nanoemulsion (479.1 nm) was more effective on both G + and G- bacteria than pure LEO. Yazgan et al. [15] also found that the antibacterial activity of lemon nanoemulsion (181.5 nm) against several foodborne pathogens, such as *S. aureus* and *Enterococcus faecalis*, was enhanced compared to 100 % lemon essential oil. It was speculated that essential oil in nanoemulsions had an improved physicochemical stability and dispersibility in food matrices, leading to easier access to microbes and consequently higher antibacterial potency [39].

The MIC of all coarse emulsion and GEON were 1.25 % (12.5 mg/mL), namely, the MICs of GEO in delivery systems were 0.125 % (1.25 mg/mL) as its proportion was 10 % (Fig. 3C). This was reasonable since double agar dilution method can not distinguish the subtle difference of MICs well. The MIC of GEON was comparable with other nanoemulsions preparing from thyme (1.56–25 mg/mL) [27] and sage essential oil (6.25–25 mg/mL) [40] against food pathogens and fish spoilage microorganism. Garzoli et al. [38] also found that the MIC of *Lavandula × intermedia* essential oil in nanoemulsion was 0.37 % against *Escherichia coli*.

Moreover, a bactericidal test was conducted to compare the anti-MRSA activity of coarse emulsion and GEON. As shown in Fig. 3D, 1MIC coarse emulsion treatment resulted in a 1.96 log reduction of MRSA *P*-1, while higher values of 2.19, 2.25 and 2.60 log were detected when cells were treated with GEON resulting from 1, 5 and 10 min ultrasonication, respectively. Moreover, the values were further increased to 2.56, 2.82, 3.21 and 3.41 log for coarse emulsion and GEONs (1, 5 and 10 min) at 2MIC, respectively. It was apparent that GEON showed higher anti-MRSA activity than coarse emulsion at the same concentration, and in the range of the tests (from 0 to 10 min), the longer the ultrasonic time, the smaller the particle size and the higher the anti-MRSA activity. The result was in line with El-Sayed et al. [41], who found that GEO in water-based delivery system with smaller particle sizes (such as microemulsions) showed higher antibacterial ability than that with larger particle sizes (such as emulsions) at the same concentration.

Furthermore, our findings were in accordance with the results regarding clove essential oil nano-emulsions [16], thymus daenensis essential oil nanoemulsion [13], and the nano-emulsion films formulated with hazelnut meal protein and clove essential oil [19]. It is reported that the partial size of nanoemulsion is pivotal in determining the antimicrobial ability of agents in the delivery system. As ultrasonic time prolonged, increased acoustic energy resulted to reduced particle size and raised specific surface area of GEO in the nanoemulsion system, thus leading to increased exposure of GEO to microbial membrane and enhanced antibacterial activity. Therefore, ultrasonic emulsification is an effective formulation strategy to exert and improve the bacteriostatic effect of GEO.

### 3.4 Growth curve analysis

**Fig. 4:**
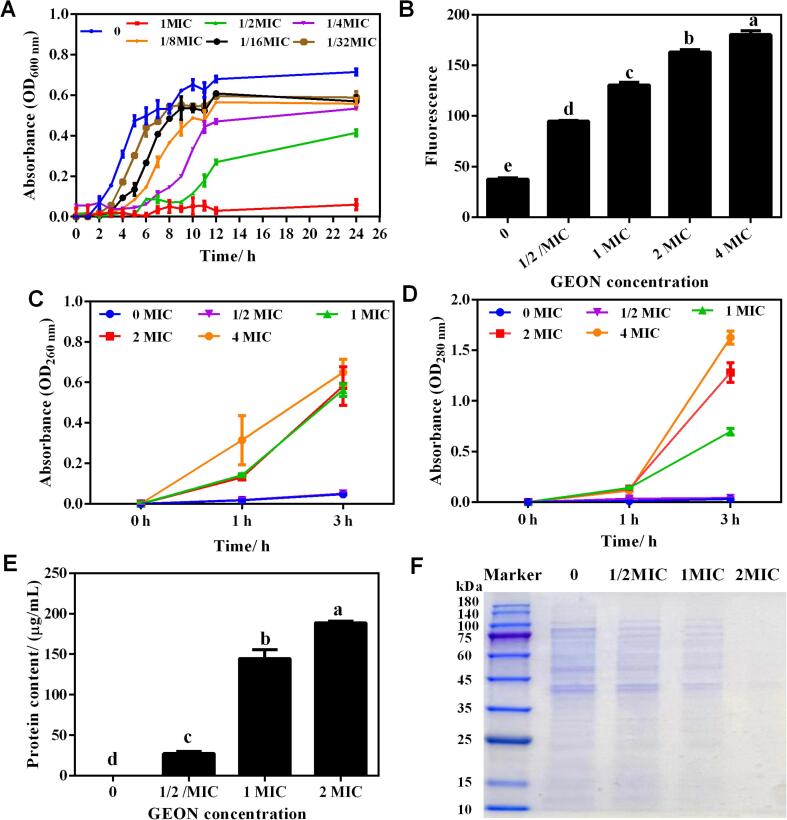
Effect of GEON (10 min) on MRSA *P*-1 growth and cellular components.A, growth curve of *P*-1 cultured in TSB with different concentration of GEON. B, cell membrane potential. C-D, extracellular DNA and protein content, detecting at OD_260 nm_ and OD_280 nm_, respectively. E, extracellular protein content, detecting using BCA protein quantification kit. F, change of intracellular soluble protein. Different lowercase letters indicate significant differences (*p* < 0.05).

Based on the above results, GEON produced by 10 min ultrasonication was further analyzed for its anti-MRSA mechanism. The effect of GEON on the growth of MRSA *P*-1 was firstly investigated, and results were shown in Fig. 4A. GEON at 1MIC could completely inhibit the growth of MRSA, and subinhibitory concentration (1/32–1/2MIC) of GEON showed a dose-dependent inhibition effect against MRSA *P*-1, as a more prolonged lag phase and a lower maximum optical density was detected when GEON concentration increased.

### 3.5 Membrane potential analysis

Resting membrane potential is one parameter that affects the cell antibiotic uptake and bactericidal action, which is therefore vital for living cells [42]. DiBAC_4_(3), a membrane potential sensitive fluorochrome, is widely used in membrane potential determination. It could enter the depolarized cell and subsequently bind to the intracellular hydrophobic sites, thus leading to increased fluorescence intensity. Conversed changes will be found in hyperpolarized cells [43]. In our study, a significant increase in fluorescence was detected in MRSA *P*-1 cells treated with GEON, which was directly proportional to the increase in membrane potential. Moreover, the membrane potential increased gradually as the increase of GEON concentration from 1/2 MIC to 4MIC. Shi et al. [44] found that *Cronobacter sakazakii* treated with lipoic acid displayed rapid cell membrane depolarization. Lou et al. [45] also reported a change of membrane depolarization in *Shigella dysenteriae* and *Streptococcus pneumoniae* cells after exposing to chlorogenic acid. Membrane depolarization is one critical type of membrane damage, and it occurs mainly due to the abnormal release of potassium and other ions [46].

### 3.6 Extracellular nucleic acids and protein

Cellular nucleic acids and protein are essential cytoplasmic constituents of microorganisms, and their release could be considered as a sign of cell membrane structure disruption. As shown in Fig. 4C, a larger content of nucleic acids was released from cells subjected to GEON treatments compared to the control. Moreover, the release of nucleic acids was in a concentration- and time-dependent manner, as GEON concentration increased from 1/2MIC to 4 MIC and treatment time prolonged from 0 to 3 h.

The released protein contents of cells subjected to GEON treatments were much higher than control, and extracellular protein content also increased in a concentration- and time-dependent manner (Fig. 4D). Quantitative analysis showed that extracellular protein content in control was nearly zero after 3 h treatment. However, the values increased significantly to 27.43, 144.68 and 188.77 μg/mL, respectively, when cells were treated with different concentrations of GEON (1/2MIC, 1MIC and 2MIC), suggesting the extensive leakage of cellular protein under GEON treatment (Fig. 4E). Meanwhile, the changes of intracellular soluble protein were investigated using SDS-PAGE to evident the effect of GEON on MRSA protein. As shown in Fig. 4F, MRSA *P*-1 cells had various soluble proteins with molecular weights ranging from 10 to 180 KDa. When cells were subjected to GEON treatments (1/2MIC-2MIC), the robustness of protein bands, especially at 35–100 KDa, decreased gradually, further suggesting the loss of intracellular protein.

The considerable release of nucleic acids and protein was a piece of strong evidence that GEON caused gross and irreversible damage to the cell membrane [23]. Previous studies have also reported the loss of membrane permeability and leakage of cytoplasmic substances in cells treated with bacteriostatic agents. Guo et al. [23] reported the releases of nucleic acids and protein from *E. coli* cells exposed to thyme essential oils nanoemulsion. Lv et al. [47] found that essential oils treatment resulted the release of cell constituents from *Saccharomyces cerevisiae*, *E. coli*, *S. aureus*, and *Bacillus subtilis* cells. The enormous loss of cellular components will inevitably result in defective and hampered intracellular metabolic processes, and finally, cell death.

### 3.7 LSCM and SEM observation

**Fig. 5:**
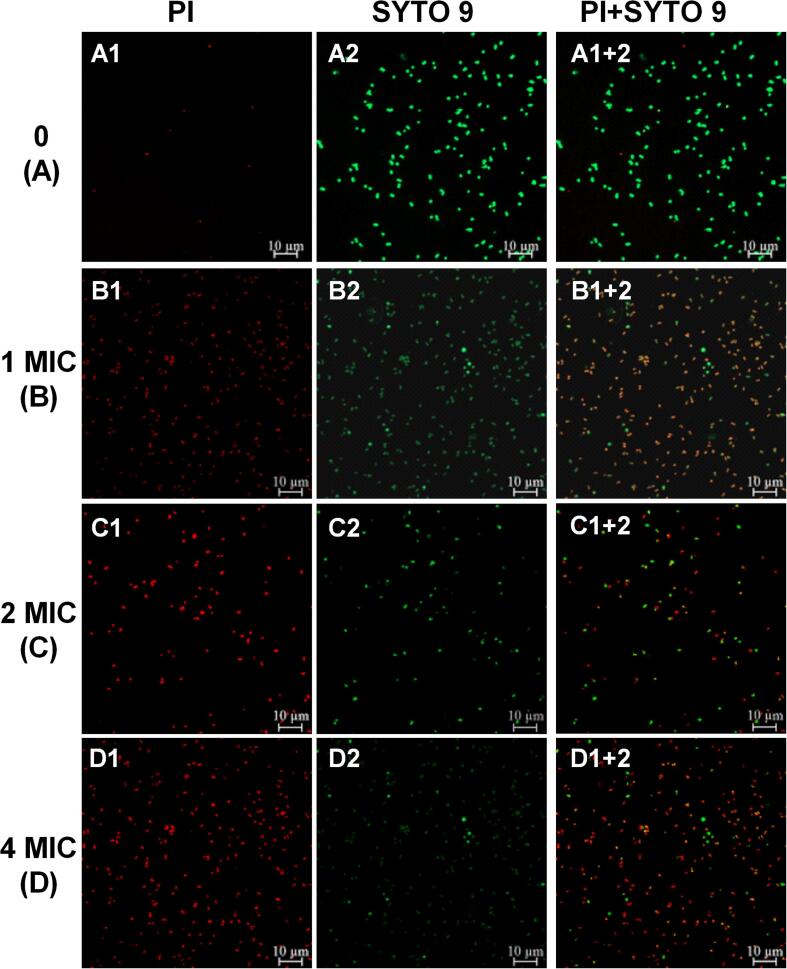
LSCM photographs of MRSA *P*-1 cells.A, control. B-D, MRSA cells treated by 1 MIC, 2MIC and 4MIC GEON (10 min), respectively.

LSCM observation combined with fluorescence dyes is useful for detecting the permeability and integrity of cell membrane. SYTO9 and PI dyes can differ the viable and dead cells, by which viable cells with intact membrane stained green fluorescent (SYTO9), and dead cells with damaged membranes stained red fluorescent (PI). As shown in Fig. 5A, the majority of MRSA cells in the control sample emitted green fluorescent, indicating the viable cells with integrity membrane. While a sharp increase of red fluorescence was detected in cells treated with 1MIC GEON (Fig. 5B). As GEON concentration increased to 2MIC and 4MIC, more red fluorescence was observed, meanwhile green fluorescence decreased accordingly (Fig. 5C and D), suggesting the destructive cell membrane and reduced viable cell. Our results were in accordance with the reports regarding the antibacterial effect of thyme essential oils nanoemulsion [23] and *Litseacubeba* essential oil [48], which demonstrated that essential oil and its nanoemulsion could alter the permeability and damage the integrity of cell membrane..

**Fig. 6:**
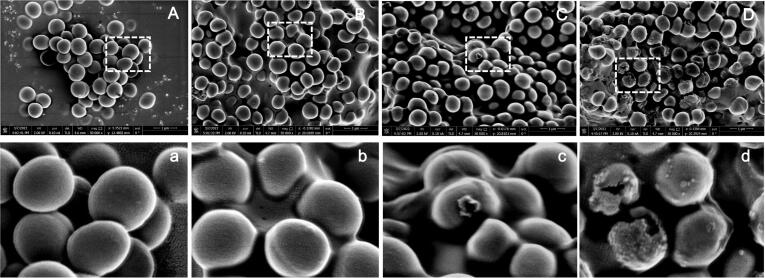
SEM photographs of MRSA *P*-1 cells. A and a, control. B and b, C and c, D and d, MRSA cells treated by 1 MIC, 2MIC and 4MIC GEON (10 min), respectively.

SEM observation was applied to visualize the morphological alterations in MRSA cells. *P*-1 cells in control sample displayed a spherical shape and smooth intact surface (Fig. 6A and a). However, a deformed and shrunken shape was detected in cells subjected to 1MIC GEON, meanwhile cell aggregation occurred accompanied with the appearance of pastelike substances (Fig. 6B and b). As GEON increased to 2MIC, the shape deformation and cell aggregation were further aggravated, leading to the appearance of cellular fragments (Fig. 6C and c). When GEON was 4MIC, the cell membrane was irreversibly destroyed and cell structure was wholly collapsed, resulting the release of cellular contents (Fig. 6D and d). These results were in line with previous SEM observations by Guo et al. [23], who visualized the disintegration of cell structure and the formation of cellular fragments and debris in *E. coli* O157:H7 treated by thyme essential oils nanoemulsion. In another study, Bhargava et al. [49] reported that oregano oil nanoemulsion treatment led to a similar morphology alternation in three foodborne pathogenic bacterias cells (*Listeria monocytogenes*, *Salmonella typhinurium*, and *E. coli* O157:H7). These results suggested that the existence of essential oils could cause severe damage to the structure, integrity and function of cell membrane.

## 4 Conclusion

Ultrasonic emulsification technique was feasible and effective in preparing nanoemulsion from GEO, and as the increase of ultrasound time, the particle size and PdI of GEON decreased significantly. GEON prepared by 10 min ultrasonication had the minimum particle size of 215 nm in the tested range, and it displayed good storage (during 30 d) and thermal stability (20-100℃), and relatively good ions stability (NaCl, MgCl_2_, and glucose). Moreover, GEON (10 min) showed the highest anti-MRSA activity among all GEONs. It exerted its antibacterial effect likely through damaging the cell membrane, as proven by the cell membrane depolarization, increased extracellular protein and DNA concentration, reduced membrane integrity, and severe morphological alterations. These results suggested that ultrasonic emulsification is a promising strategy for the deep processing and utilization of GEO, and GEON prepared by 10 min ultrasonication is potential for controlling and eliminating MRSA in livestock and poultry products.

## CRediT authorship contribution statement

**Miaomiao Liu:** Conceptualization, Formal analysis, Funding acquisition, Investigation, Project administration, Visualization, Writing – original draft, Writing – review & editing. **Yue Pan:** Investigation, Data curation, Methodology, Software. **Mingxing Feng:** Formal analysis, Software, Writing – review & editing. **Wei Guo:** Investigation, Data curation, Methodology. **Xin Fan:** Formal analysis, Methodology, Validation. **Li Feng:** Project administration, Validation. **Junrong Huang:** Supervision, Resources. **Yungang Cao:** Project administration, Resources, Supervision, Writing – review & editing.

## Declaration of Competing Interest

The authors declare that they have no known competing financial interests or personal relationships that could have appeared to influence the work reported in this paper.

## Data Availability

Data will be made available on request.
